# Protocol for an evaluation of adherence monitoring and support interventions among people initiating antiretroviral therapy in Cape Town, South Africa—a multiphase optimization strategy (MOST) approach using a fractional factorial design

**DOI:** 10.1186/s13063-023-07322-z

**Published:** 2023-05-05

**Authors:** Lauren Jennings, Rebecca L. West, Nafisa Halim, Jeanette L. Kaiser, Marya Gwadz, William B. MacLeod, Allen L. Gifford, Jessica E. Haberer, Catherine Orrell, Lora L. Sabin

**Affiliations:** 1 Desmond Tutu Health Foundation, Institute of Infectious Diseases and Molecular Medicine and the Department of Medicine, University of Cape Town, Cape Town, South Africa; 2 Department of Global Health, Boston University School of Public Health, Boston, MA USA; 3 Silver School of Social Work, New York University, New York, NY USA; 4 Health Economics and Epidemiology Research Office, Department of Internal Medicine, School of Clinical Medicine, Faculty of Health Sciences, University of the Witwatersrand, Johannesburg, South Africa; 5 Section of General Internal Medicine, Department of Medicine, Boston University School of Medicine, 72 E Concord Street, Boston, MA 02118 USA; 6 Center for Healthcare Organization and Implementation Research, VA Boston Healthcare System, 150 S. Huntington Ave, Boston, MA 02130 USA; 7 Department of Health Policy and Management, Boston University School of Public Health, Talbot Building, T348W, Boston, MA 02118 USA; 8 Center for Global Health, Massachusetts General Hospital, Boston, MA USA; 9 Department of Medicine, Harvard Medical School, Boston, MA USA

**Keywords:** HIV, Antiretrovirals, Medication adherence, Electronic adherence monitoring, Behavior change, MOST framework

## Abstract

**Background:**

South Africa bears a large HIV burden with 7.8 million people with HIV (PWH). However, due to suboptimal antiretroviral therapy (ART) adherence and retention in care, only 66% of PWH in South Africa are virally suppressed. Standard care only allows for suboptimal adherence detection when routine testing indicates unsuppressed virus. Several adherence interventions are known to improve HIV outcomes, yet few are implemented in routinely due to the resources required. Therefore, determining scalable evidence-based adherence support interventions for resource-limited settings (RLS) is a priority. The multiphase optimization strategy (MOST) framework allows for simultaneous evaluation of multiple intervention components and their interactions. We propose to use MOST to identify the intervention combination with the highest levels of efficacy and cost-effectiveness that is feasible and acceptable in primary care clinics in Cape Town.

**Methods:**

We will employ a fractional factorial design to identify the most promising intervention components for inclusion in a multi-component intervention package to be tested in a future randomized controlled trial**.** We will recruit 512 participants initiating ART between March 2022 and February 2024 in three Cape Town clinics and evaluate acceptability, feasibility, and cost-effectiveness of intervention combinations. Participants will be randomized to one of 16 conditions with different combinations of three adherence monitoring components: rapid outreach following (1) unsuppressed virus, (2) missed pharmacy refill collection, and/or (3) missed doses as detected by an electronic adherence monitoring device; and two adherence support components: (1) weekly check-in texts and (2) enhanced peer support. We will assess viral suppression (<50 copies/mL) at 24 months as the primary outcome; acceptability, feasibility, fidelity, and other implementation outcomes; and cost-effectiveness. We will use logistic regression models to estimate intervention effects with an intention-to-treat approach, employ descriptive statistics to assess implementation outcomes, and determine an optimal intervention package.

**Discussion:**

To our knowledge, ours will be the first study to use the MOST framework to determine the most effective combination of HIV adherence monitoring and support intervention components for implementation in clinics in a RLS. Our findings will provide direction for pragmatic, ongoing adherence support that will be key to ending the HIV epidemic.

**Trial registration:**

ClinicalTrials.gov NCT05040841. Registered on 10 September 2021.

**Supplementary Information:**

The online version contains supplementary material available at 10.1186/s13063-023-07322-z.

## Background

Advances in antiretroviral therapy (ART) have reduced illness and death for people with HIV (PWH), but major gaps in the care continuum persist. Sub-Saharan Africa is home to nearly 70% of the world’s PWH; South Africa, with 7.8 million PWH, bears the continent’s greatest HIV burden. By 2018, nearly five million PWH had accessed ART in South Africa, but weaknesses were clear: 17% of PWH who start ART fall out of care by 16 weeks and >20% are lost in the first year [1–3]. Adherence to ART in South Africa ranges from 40 to 75% [3–5], far below what experts believe is required for successful treatment (typically at least 80%, even with potent, modern regimens) [6–8]. Mainly due to suboptimal adherence and retention, only 51–86% of people show suppressed virus at 12 months [3, 4, 9–12]. Overall, approximately 66% of PWH are believed to be virally suppressed [13]. These outcomes predict higher mortality, more HIV transmission, and drug-resistant HIV, hindering the World Health Organization’s “End HIV/AIDS by 2030” goals [14].

Early detection of suboptimal adherence among PLW initiating ART, and linkage to support for these patients is critical. The evidence shows that people who miss doses early in treatment go on to miss clinic visits, exhibit poor outcomes, and be disproportionately lost to care [15–17]. Existing data from studies conducted in the City of Cape Town show that PWH with unsuppressed virus (>1000 copies/ml), potentially indicating suboptimal adherence, were more likely to be lost to care in later years than virally suppressed patients [18, 19]. Intervention studies provide ample evidence that patients can benefit from support interventions [20], including peer groups [21], motivational interviewing [21–23], and text message reminders [21, 23, 24]. In addition, there is evidence suggesting that multi-component interventions provide stronger support than single-component interventions [23, 25]. However, such interventions are often not implemented in routine care due to cost and resource requirements that influence systems of care. Moreover, people who will need extra support are not routinely identified at the time of ART initiation: a key challenge is that new patients may struggle and be lost to care before they are identified as being in need of support.

There are several methods of adherence monitoring and ways of identifying people who need extra support—each with its own strengths and weaknesses. The current standard for monitoring adherence in South Africa is through self-report, which is often unreliable [2], and through infrequent viral load measurements, recommended at 4 and 12 months after initiating ART and annually thereafter [26]. However, feedback of the viral load result to the patient is often only given at the next routine clinic visit, resulting in a delay in initiating adherence support measures. Electronic adherence monitors (EAM) that track adherence in real time are acceptable and feasible in many settings and have been used to deliver reminders and trigger just-in-time support [27–32]. In HIV treatment, the benefit-cost ratio of broad use of EAM in clinical settings is unknown. Another method that leverages existing infrastructure is pharmacy refill monitoring (PRM). PRM data can detect suboptimal adherence after a missed refill, prior to viral load becoming unsuppressed [2, 33]. A third approach is early patient outreach when unsuppressed virus is detected. Early outreach is not done in most resource-limited settings, but is feasible and well-liked by patients [34].

This study’s primary research goal is to identify the optimal combination of evidence-based and scalable HIV interventions for resource-limited, high-burden settings. In Cape Town, health officials have established Risk of Treatment Failure (ROTF) clinics, where patients with unsuppressed virus receive extra support via (a) one nurse-led counselling session and (b) peer counselling (3–4 sessions) [35]. Our hypothesis is that the test we have designed will allow us to identify a superior combination of interventions. Thus, working in collaboration with the City of Cape Town, we plan to (1) test the relative contributions of five promising intervention components, three comprising adherence monitoring methods, and two comprising extra support elements; (2) collect cost and other implementation data; and (3) optimize a multi-component intervention package with high levels of cost-effectiveness and characteristics identified as necessary for implementation success.

The gold standard for testing interventions is the randomized controlled trial (RCT); however, when planning and testing an intervention with multiple components, paired comparisons of the individual elements is complicated and costly. A more efficient approach to developing a multi-component intervention is the novel multiphase optimization framework (MOST) [36, 37], an engineering-inspired framework for identifying the most efficacious combination of components that can then be included in a “packaged” or multi-component intervention. In this study, SUSTAIN (Supporting Sustained HIV Treatment Adherence after Initiation), we will employ a MOST framework to determine the combination of interventions—with the highest levels of efficacy and cost-effectiveness and that are feasible and acceptable in primary care clinics—compared to standard of care.

## Methods

**Fig. 1:**
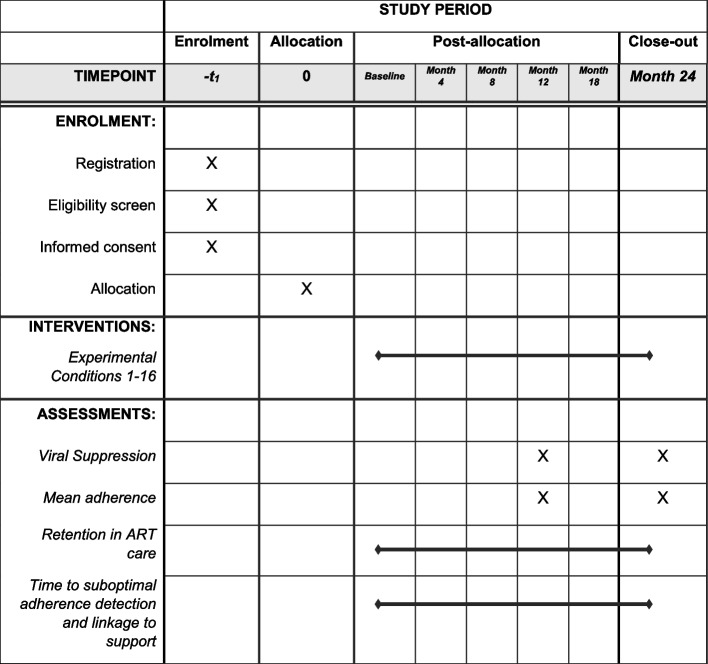
Schedule of enrolment, interventions, and assessments

The study protocol has been reported in accordance with the Standard Protocol Items: Recommendation for Clinical Intervention Trials (SPIRIT) guidelines [38] (see Fig. 1 and Additional File 1 for details).

### Overview

MOST has three phases: (1) preparation; (2) optimization; and (3) evaluation [39]. The preparation phase involves the development of a conceptual framework, the identification of feasible candidate intervention components, and setting the optimization objective. In the optimization phase, comparative effectiveness of the individual components is assessed using one of several potential study designs and, finally, in the evaluation phase the optimized intervention package is assessed through an RCT.

#### Conceptual framework

**Fig. 2:**
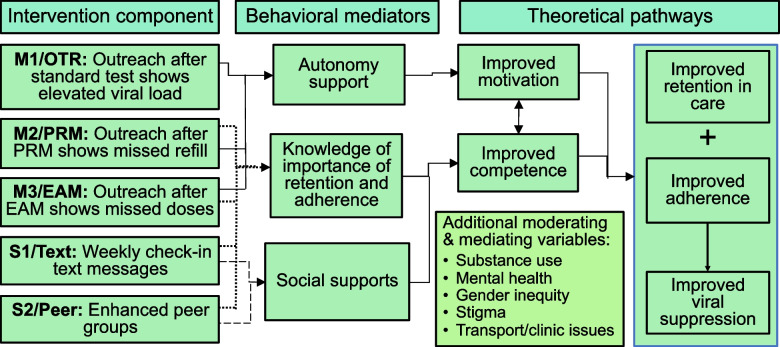
Theoretical framework of the SUSTAIN study. Abbreviations. OTR, outreach. PRM, pharmacy refill monitoring. EAM, electronic adherence monitoring

Our overall study is informed and supported by an adapted version of the Self-Determination Theory (SDT). SDT posits that a person is motivated to alter behaviors when their needs for connectedness, efficacy, and autonomy have been satisfied (see Fig. 2) [40, 41]. We hypothesize that purposeful adherence monitoring and outreach when suboptimal adherence occurs, regardless of specific form of monitoring, will increase feelings of autonomy, prompting improved motivation and competence. We further hypothesize that active adherence monitoring alongside strengthened adherence support will increase patients’ appreciation for the value of adherence and retention and promote their competency. Finally, we adapted the model to include “social support” as a mediator to improve competency and adherence (as reflected in other social support theories and Social Cognitive Theory) [42, 43]. This adapted model also accounts for other major variables such as substance use, mental health, and stigma, which are known to influence ART outcomes directly and as moderating factors [44–49].

Prior to beginning this study, the team carried out the preparation phase of MOST. We partnered with local officials and clinical staff in Cape Town to review evidence for adherence interventions and conduct formative research to identify the most effective, acceptable, and feasible intervention components for patients and providers. These components formed the basis for a pilot study [50] in which we used in-depth interviews (IDIs) with patients and focus groups with providers to determine the most preferred intervention components to advance to the next phase. For our final study design for the optimization phase, we incorporated the two most preferred support strategies identified in our pilot study: (a) weekly check-in texts (S1); and (b) enhanced peer support, using motivational interviewing (MI) (S2). We also included three means of rapidly identifying and reaching out to nonadherent patients: (1) Early call to the patient (e.g., within a week) after a standard viral load test showing an unsuppressed virus (M1); (2) patient pharmacy refill monitoring, with an early call to the patient after a missed refill (M2); and (3) real-time EAM, again with an early call to the patient if doses are missed (M3). These five intervention components are described in Table 1.

**Table 1:** Intervention components

**Component**	**Component type**	**Frequency**	**Method of delivery**
Standard of Care (SoC): Informing the patient of the VL result at their next clinic visit and initiating counselling	SoC	At the next visit after month 4 and month 12 after ART initiation	In-person at the clinic
M1: Call to the patient after unsuppressed VL on routine testing	Adherence monitoring	At month 4 and month 12 after ART initiation	Direct phone calls
M2: Pharmacy refill monitoring and call to the patient	Adherence monitoring	Monthly or 2 monthly for 12 months, depending on clinic visit schedule	Direct phone calls
M3: Electronic adherence monitoring and call to the patient	Adherence monitoring	Reviewed weekly	Direct phone calls
S1: Weekly check-in text messages	Adherence support	Weekly for 12 weeks once suboptimal adherence detected	Automated weekly text messages with option to reply
S2: Peer support (Basic or enhanced)	Adherence support	Monthly for 3 months once suboptimal adherence detected	Basic: One-on-one or group adherence sessions delivered by clinic counsellors Enhanced: One-on-one motivational interviewing delivered by study staff

Abbreviations: *SoC*, standard of care; *VL*, viral load; *ART*, antiretroviral therapy

#### Setting the optimization objective

The optimization objective is to identify the combination of interventions with the greatest levels of efficacy (viral suppression and retention in care) and cost-effectiveness that are feasible and acceptable in primary health clinics supplying ART in resource-limited settings.

### Optimization phase

This phase of the study—SUSTAIN—involves a fractional factorial design and regression analysis adjusted for clustering by clinic to accomplish three aims: (1) to determine the relative efficacy of five intervention components on the primary and secondary outcomes (described below); (2) to assess implementation, service, and client outcomes pertinent to each intervention component; and (3) to model the multi-component intervention optimized for cost-effectiveness and implementation success.

#### Study setting

This study is taking place in three City of Cape Town clinics in the Klipfontein and Mitchell’s Plain health districts in South Africa. Each of these clinics serves a population with a high prevalence of HIV. Although precise numbers are not available, prevalence in these districts is believed to be higher than in Cape Town generally which was estimated to be 9.5% in 2017 [51]. Each clinic provides free ART services delivered by doctors, clinical nurse practitioners, and registered nurses [26].

#### Eligibility criteria

Study participants must be adolescents aged 16–17 years or adults ≥18 years of age, who are presenting to the clinic for initiation of ART. Study participants must be willing and able to sign informed consent or, in the case of minors, informed assent with parents willing to sign informed consent. They must have a working cellphone and be willing to receive study-related text messages. They must also be willing and able to comply with study procedures, including using an EAM and providing current contact information. We aim to enroll 512 participants (roughly 170 per clinic) between March 2022 and February 2024.

#### Recruitment

Participants will be recruited by community research workers who are embedded within the clinics and work closely with clinic staff who are initiating people on ART. Study leadership will evaluate enrollment rates on a monthly basis to identify if there is a need to adjust the recruitment strategy.

#### Informed consent

All potential participants in the trial will undergo informed consent procedures, including emphasis that participation is voluntary and that refusal will have no negative impacts on the ART service they receive at their clinic. Adult eligible participants aged 18 years and over who agree to enroll in the study will provide full written informed consent. Eligible adolescents of ages 16 and 17 years will not be able to enroll unless they are willing to complete an assent form and are able to bring a parent or guardian with them to complete a parental full informed consent document. All informed consent and assent processes will be conducted in the language of the participants choice (usually English or Xhosa); and the forms will be available in these languages as well. The informed consent and assent document will contain contact details for study staff who are available to answer questions about the study.

#### Timeline

Participants will be enrolled for a total of 24 months, with study follow-up visits at months 4, 8, 12, 18, and 24 (Fig. 1). The intervention components will be applied for the first 12 months of enrollment.

#### Intervention components

At enrollment, each participant will be issued an EAM to measure adherence over time, and for those assigned to M3, to actively monitor adherence. Enrolled participants will be randomized to one of 16 study conditions (i.e., combination of monitoring and intervention strategy—see Table 2). Four of the five intervention components (M1, M2, M3, & S1) can either be switched “on” (the component is applied) or switched “off” (the component is not applied). The counselling component (S2) can be either basic (standard of care) or enhanced (motivational interviewing). As this study includes only behavioral interventions, all routine medical care provided by the clinics will continue as needed. There is no prohibited concomitant care. While the study interventions monitor and promote adherence and retention in care, there are no additional measures to promote retention within the study itself (e.g., outreach to participants who have missed study visits), as these additional activities would influence retention in care as an outcome and undermine the assessment of our interventions.

**Table 2:** Intervention components and study conditions in the fractional factorial design

**Study condition** **Intervention components**	1	2	3	4	5	6	7	8	9	10	11	12	13	14	15	16
Core (standard care): After a VL test shows unsuppressed virus, the patient is alerted at the next clinic visit and given a counselling session	X	X	X	X	X	X	X	X	X	X	X	X	X	X	X	X
M1. Call to the patient after VL test result of unsuppressed virus (M1/Call)	O	O	O	O	O	O	O	O	X	X	X	X	X	X	X	X
M2. Pharmacy refill monitoring + Call to the patient (M2/PRM)	O	O	O	O	X	X	X	X	O	O	O	O	X	X	X	X
M3. Electronic adherence monitoring + Call to the patient (M3/EAM)	O	O	X	X	O	O	X	X	O	O	X	X	O	O	X	X
S1. Weekly check-in text messages (S1/Text)	O	X	O	X	O	X	O	X	O	X	O	X	O	X	O	X
S2. Peer support counselling: Basic (B) or Enhanced (E) (S2/Peer)	E	B	B	E	B	E	E	B	B	E	E	B	E	B	B	E

Abbreviations: *VL*, viral load; *PRM*, pharmacy refill monitoring; *EAM*, electronic adherence monitoring

#### Outcome measures

We will be collecting data using viral load results (at month 4 and month 12—obtained from patient records and the National Health Laboratory Service online system), EAMs (daily), pharmacy refills (monthly), and participant and staff surveys. The primary outcome will be viral suppression at month 24 post-enrollment as measured by plasma HIV-1 viral load <50 copies/mL. Secondary outcomes will include viral suppression at month 12, change in viral load over time, and total days of unsuppressed virus during study participation. Additional secondary outcomes include the following: mean electronic adherence and percent of participants ≥90% and ≥80% adherence in months 12 and 24; mean adherence over months 1–12 and 1–24; retention in ART care (percent of participants attending all refill visits within 7 days for monthly refills and 14 days for longer refill intervals) over months 1–12 and 1–24; percent of participants attending ≥75% refill visits (within 7 days), over months 1–12 and 1–24; percent of participants lost to care, defined as no clinic contact for ≥12 weeks at month 24); and time to suboptimal adherence detection and linkage to support (number of days from month 0 to day identified as nonadherent, as per the definition used in their intervention; number of days from month 0 to linkage to ROTF support).

#### Mediator and moderator variables

Annual surveys will allow us to collect data on SDT constructs (such as autonomy support, motivation, perceived self-competence, social support, and ART knowledge) to test the effect on each in mediating the association between the intervention and outcomes. Further, we will collect data on substance use, depression, gender inequity, stigma, and transport or clinic-related issues to test the extent to which each of these variables independently or in combination moderate the association between the intervention and outcomes.

#### Implementation outcomes

**Fig. 3:**
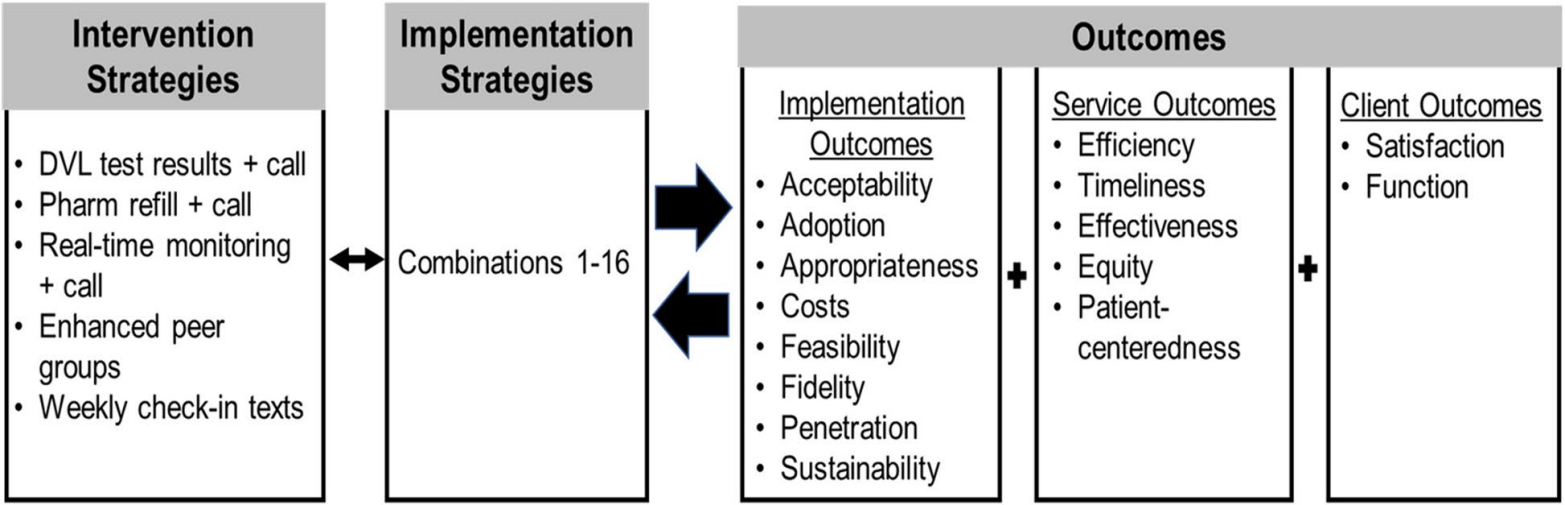
Modified Proctor Framework [52]. Abbreviations. Abbreviations. DVL, detectable viral load

Translation of the optimized intervention into real-world benefits will require a clear understanding of the implementation process. We will assess implementation using a modified Proctor framework (Fig. 3) [52], as this enables measurement of the distinct implementation outcomes in relation to the intervention outcomes, while also considering impact on clients. Brief questionnaires will be administered to all enrolled patients and clinic staff by the study team at two time points during the study to assess both short-term and long-term experiences. A subset of study participants (*n*=30) will also be selected for IDIs to explore acceptability, intervention appropriateness, feasibility, and patient satisfaction further. Clinic observations at each study site will be conducted to assess the fidelity of intervention implementation.

#### Costing

A micro-costing analysis will be undertaken to determine the cost of implementing SUSTAIN intervention components and their combinations. Micro-costing is widely used in costing studies [53–55]; it is a bottom-up approach used to estimate the cost of setting up and delivery of an intervention, involving collecting detailed information about the resources required to implement an intervention, and assigning economic unit costs to each component of resource use.

Costs collected will include set-up or non-recurrent unit costs (such as training staff or purchasing materials) and recurrent costs (such as staff salaries). Costs related to research activities will be excluded. We will estimate costs in South African Rand and derive totals in United States Dollars. Intervention costs collected will include equipment, supplies, and services (e.g., text messaging) in accordance with an integrated framework based on the Eaton framework [56] for HIV service delivery and the Saldana framework [57] for costing intervention implementation.

Structured costing forms (log sheets) will be developed and distributed to the study manager and SUSTAIN study staff. During designated periods, study staff will complete individual log sheets to record how much time is spent training each participant to use their EAM devices and conducting counselling. The study manager will also regularly consult with the study coordinator, data clerks/managers, drivers, and other individuals conducting SUSTAIN intervention activities to capture time and costs incurred. We will collect data at multiple time points: pre-study start to collect intervention set-up costs, the first 4 months of study start to capture start-up time and costs, and for an additional 1–2 months in study year 1 and study year 2 to capture steady state costs. Two study investigators will conduct one 2-week time and motion study at each participating clinic once study activities are operating at a steady state to evaluate time needed to conduct each intervention component; these observational data will be used to validate and/or adjust time estimates captured through study staff self-report. All time and cost data will be used to complete intervention cost sheets.

#### Sample size

We anticipate we will need 512 participants for this study to ensure the power to detect a clinically meaningful individual effect of intervention components on HIV viral suppression. Our sample size of 512 combines two estimates: (a) the number of individuals needed to detect a clinically significant sized effect in HIV viral suppression rate due to receipt of an intervention component; and (b) the number of individuals who we may lose over the course of the study. To elaborate, first, we assume we will have a 14-percentage point increase in viral suppression in participants receiving a specific intervention component compared to those who receive the standard of care alone. Our estimate of 14 percentage point increase is based on recent work at Cape Town clinics indicating suppression rates of 51–79% at month 12 post-initiation of ART [4]. Therefore, using the baseline HIV viral suppression rate of 60%, endline rate of 74%, a two-sided alpha of 0.05 and 80% power, and intraclass coefficient component (ICC) of 0.15, we calculated that we will need a sample of 458, using PS Power and Sample Size Calculations [58]. While we do not know the ICC for each clinic to inform the design stage (for the sample size calculation in a RCT design involving participants representing three clinics), we conservatively assumed they are 0.15, based on recent studies showing ICC estimates ranging between 0.01 and 0.12 [59–61]. To account for lost to follow-up of up to 10% (based on site experience in other recent studies), we will need to recruit a total of 510 participants, which we increased to 512 to be able to sample an equal number of participants across three clinics and 16 study conditions. For secondary outcomes, this sample size will allow us to detect differences in ≥90% adherence and in retention of 11–15%, a meaningful range that is reasonable given other studies [22, 62].

#### Blinding

This is an unblinded study as the study condition to which each participant has been assigned can be deduced by looking at the interventions that they have received. We considered conducting a blinded analysis. However, since all the information to unblind the assigned groups would be required, we determined that there was no way of practically conducting a blinded analysis and determined that all the analyses will be unblinded.

#### Randomization

We will randomly assign 512 participants representing three clinics to one of 16 study conditions (Table 2) using a permutation allowing for the study conditions to include an equal number of participants, which is 32 (16*32 = 512). We developed the random assignment of participants in Stata v.14 using *runiform* and *rank* functions. The randomization sequence is concealed from all study staff involved in recruitment, enrollment, and randomization. Once eligibility has been determined, designated study staff will randomly assign participants to a study condition, using the REDCap electronic database.

#### Data and statistical analysis

Study staff will collect data directly onto the REDCap database using password-protected tablets. The REDCap database was designed with automated internal checks to ensure data entered are accurate and complete. In addition, the site follows a data management plan, which outlines quality control and quality assurance practices. All participants will be assigned a study identifier and data for analysis will only be identified through this number. All documents that contain personal identifiers will be kept in locked cabinets with limited access.

We will employ a series of descriptive and inferential statistical methods and techniques for data analysis. For dataset preparation, missing baseline data on socio-demographics will be imputed using a single imputation technique if the proportion missing is <10% or using a multiple imputation technique if the proportion missing is ≥10%, respectively. In cases of missing viral load data, we will treat the result as detectable, in accordance with our primary intention-to-treat (ITT) analytic approach, similar to other studies [3, 63]. For missing adherence data, the most recent month’s adherence will be used to estimate single-month adherence; for cumulative calculations, available data over the period will be used.

To estimate treatment effects, we will adopt an ITT analytic approach and all 512 participants will be analyzed according to their randomized condition. The primary outcome is viral suppression at 24 months (see Table 1), measured as a binary variable, to allow us to assess a sustained post-intervention effect [64]. We will use logistic regression to estimate main and interaction effects on the odds of viral suppression. We will use an exchangeable correlation matrix, accounting for clustering by health facility for all analyses. Participants’ receipt of intervention components will be effect-coded. That is, following Kugler et al., [65] we will code participants’ receipt of an intervention component as 1, and nonreceipt as −1. To estimate the main effect of an intervention component, we will multiply the coefficient term by two and exponentiate it (i.e., implementing the mathematical operation as follows: Exp(2*coefficient term)). We will use the same approach to estimate interaction effects between components. Similarly, we will use logistic regression to estimate effects of components on secondary outcomes measured as a binary outcome (viral load at month 12; adherence; retention). We will use linear or Poisson regression to estimate effects of components on outcomes measured as a continuous variable (e.g., mean change in viral load from month 0 to 12 and month 0 to 24) or count variable (e.g., days of unsuppressed virus), respectively. We will compare dropout rates and characteristics of participants who drop out or are lost to follow-up with those retained to assess potential for bias. We will also assess evidence of contamination by examining data from our detailed REDCap records and texting logs, using any such evidence when interpreting data on outcomes. Moderating effects will be explored through regression modeling following Hayes [66]; mediators will be assessed using the approach by Valeri and VanderWeele, which allows for logistic modeling, and sensitivity analyses will be conducted on key assumptions per Imai [67, 68]. For all quantitative analyses, we adopted REDCap for data capture and SAS for data analysis [69].

IDIs will be analyzed for potential mediating and moderating influences over time (e.g., reductions in substance use and stigma) using content analysis [70], involving iterative transcript review, label development, creation of operational definitions, and codebook development. We will doubly code ~20% of interviews and discuss discrepancies to achieve consensus. After codebook completion, transcripts will be coded in Dedoose (version 8.3.11). We will identify direct statements to illustrate findings.

#### Cost-effectiveness analysis

We will identify the intervention components shown to be efficacious, taking main effect sizes and interactions into account. Estimates on durability of effect will be conservative for the main analyses (e.g., we will assume the same effect over 24 months as observed in the study). The main cost-effectiveness analyses will assume a payer perspective and a 3% discount rate. Sensitivity analyses will explore the impact of uncertainty on key variables, varying the probability distributions of each factor as well as the time horizon. We will consider 5-year and 10-year time horizons, as well as different perspectives (payer and societal) and examine the effect of different discount rates (0%, 5%). Further, we will undertake cost-effectiveness analyses guided by the Proctor framework [52]. Thus, we will determine an adjusted CE outcome (AdCE) for each intervention component as a function of CE plus implementation and client outcomes defined in Aim 2. We will apply utility levels obtained from key stakeholders, incorporating the value they attach to each implementation outcome, and calculate AdCEs using the following equation:

$$AdCE = CE / ({IO}_{1}{U}_{1} + \dots + {IO}_{8}{U}_{8})$$

where CE = cost-effectiveness, IO is the success score of specific outcomes, and U is the utility of each outcome. Utilities will be expressed dichotomously (high value = 1; low value = 0) for simplicity and clarity. Thus, a given CE outcome may be more (or less) valued in the context of higher (or lower) implementation outcomes (e.g., acceptability and adoption), all other factors being similar. After the modeling exercises, members of the core team will identify the options that best combine a positive effect, low cost, and implementation priorities, including staff time required, eliminating poorly performing and costly elements.

#### Ethics approval

This study received ethical approval from the Boston University Institutional Review Board (IRB No: H-41920) on 14 September 2021 and the University of Cape Town Human Research Ethics Committee (HREC Ref: 568/2021) on 10 November 2021.

A trial management committee consisting of the principal investigators, key co-investigators, and site operations managers meets on a monthly basis to discuss study progress, monitoring, and operations.

We will convene an independent Data Safety Monitoring Board (DSMB) for this study comprised of individuals with topic and region expertise matching the study characteristics . The DSMB will periodically review cumulative study data to independently evaluate safety, study conduct, scientific validity, and data integrity of the study. The DSMB will assess interim results 12 months after 50% of the sample has been enrolled to independently determine study continuation. While no medical adverse events are expected in this study, there is the potential for social or mental harms. These will be collected spontaneously, reviewed by the trial management committee, and reported to both the DSMB and ethics committee.

#### Dissemination

Study findings will be disseminated nationally (in South Africa) and internationally through conference presentations and articles in peer-reviewed, open access journals. Additionally, results will be disseminated locally to Cape Town city officials and clinic staff through reports and workshops. De-identified participant-level data will be made available in an open access data repository after critical analyses are conducted.

The study is registered on ClinicalTrials.gov which will be maintained throughout the study. Study results will be published on ClinicalTrials.gov following study completion in accordance with NIH policy (N0T0D-16-149). All requirements from the WHO Trial Registration Data Set can be found on ClinicalTrials.gov with the exception of ethics review details which can be found within the protocol.

## Discussion

The MOST framework provides a novel study approach, which allows us to test the effects of five evidence-based intervention components in one study. Of the five intervention components, three are methods of non-adherence detection (raised viral load, missed pharmacy refill visits, missed EAM doses) plus patient outreach, and two are adherence support methods (weekly check-in texts, enhanced adherence counselling). The three monitoring methods allow for the evaluation of techniques for detecting non-adherence that increase in complexity, from a simple phone call upon detection of a raised viral load to real-time response to non-adherence through EAM. The two support elements are relatively simple and low-cost evidence-based interventions that build on standard roles in clinical practice. The enhanced counselling provided by motivational interviewing strengthens existing counselling efforts, while the text message intervention extends clinic-patient communication in a way that leverages common mobile phone usage. All five of the intervention components can be integrated into Cape Town healthcare systems and, while these will not overcome all challenges that ART patients experience (e.g., structural barriers such as food insecurity), they will represent scalable, feasible, acceptable, and effective options. Notably, they are all behavioral approaches grounded in the experience and priorities of local health officials with whom we have worked to identify scalable interventions. While the study will only be conducted in Cape Town, it may be broadly adaptable to other similar settings.

This study will provide rigorous quantitative data on the effects of the five intervention components. These data will be strengthened by the rich qualitative data to be collected from participants who are at high risk of suboptimal adherence due to the presence of social and structural barriers such as substance abuse, gender inequity, transport challenges, food insecurity, stigma, and mental health issues.

We anticipate several challenges in the implementation of this protocol. The fractional factorial design is complex and study staff will have to assess and support fidelity in the implementation of each component and to ensure that any cross-component contamination is minimized. To mitigate this risk, the study team has developed a REDCap database in which to collect participant data which alerts the user to each participant’s randomization allocation and assists in the flow of each component. The analysis of study data will also be complex and will require exploration of each intervention alone and in combination with others.

Our overriding goal is to identify feasible and effective interventions that are cost-effective and can be optimally combined and scaled up in Cape Town clinics. Ideally, doing so would involve conducting a study whereby clinic staff are themselves implementing all features of the interventions to provide a reasonable test of acceptability and feasibility. However, the heavy workload of clinic staff currently and the imposition of such an approach renders it impractical. Thus, our approach involves implementation of all intervention components by study staff. While we will collect data that can approximate how these interventions could be implemented, this study will not measure the true feasibility of each clinic to implement each component. Future work will likely be needed to evaluate implementation by clinic staff. In addition, for those randomized to receive basic counselling delivered by the clinic staff, the additional methods of suboptimal adherence detection may result in an increased number of clinic patients receiving adherence counselling in clinics, increasing the burden on clinic staff. This work may, however, be offset by the addition of study staff to provide enhanced counselling to some patients.

In sum, this study aims to use innovative methods to make progress on moving effective ART adherence interventions into clinics where they will be of help to patients. We began by identifying effective and scalable interventions from the literature and then, through further research, selected those that were acceptable to a wide range of stakeholders (health department officials, healthcare providers, and patients) [50]. Through the SUSTAIN study, we will simultaneously assess these interventions in order to provide policy makers with the best possible evidence (using both HIV-related outcomes and implementation-related outcomes) to decide which combination intervention package will most improve outcomes in patients starting ART in primary care clinics. Our findings will provide practical and feasible options for ongoing adherence support that will be a key component of efforts to end the HIV epidemic.

## Trial status

The trial is currently using protocol version 2.1 (2 June 2022). Recruitment began on 9 March 2022 and is estimated to be completed in February 2024. There are currently no plans for additional studies using these data.

## Supplementary Information

**Additional file 1.** SPIRIT Checklist for Trials.

## Data Availability

The protocol, study instruments, consent forms, and terms of reference for the DSMB described in this article can be freely and openly accessed at: https://desmondtutuhealthfoundation.org.za/admin-dthf/news/about-the-sustain-study-supporting-sustained-hiv-treatment-adherence-after-initiation/. Routine updates on the status of the trial will be provided at the above website and on ClinicalTrials.gov (NCT05040841). Questions and requests can be directed to the co-principal investigators Dr. Lora Sabin (lsabin@bu.edu) and Dr. Catherine Orrell (catherine.orrell@hiv-research.org.za).

## Abbreviations

AIDS: Acquired immune deficiency syndrome
ART: Antiretroviral therapy
DSMB: Data Safety Monitoring Board
EAM: Electronic adherence monitors
HIV: Human immunodeficiency virus
HREC: Human Research Ethics Committee
ICC: Intraclass coefficient component
IDI: In-depth interview
IRB: Institutional Review Board
ITT: Intention-to-treat
MOST: Multiphase optimization strategy
PWH: People with HIV
RCT: Randomized control trial
RLS: Resource-limited setting(s)
ROTF: Risk of treatment failure
PRM: Pharmacy refill monitoring
SDT: Self-determination theory
SUSTAIN: Supporting Sustained HIV Treatment Adherence after Initiation
